# Redox Status of Postmenopausal Women with Single or Multiple Cardiometabolic Diseases Has a Similar Response to Mat Pilates Training

**DOI:** 10.3390/antiox11081445

**Published:** 2022-07-26

**Authors:** Ana Luiza Amaral, Jaqueline Pontes Batista, Igor Moraes Mariano, Ludimila Ferreira Gonçalves, Júlia Buiatte Tavares, Adriele Vieira de Souza, Douglas C. Caixeta, Renata R. Teixeira, Erick P. de Oliveira, Foued S. Espindola, Guilherme Morais Puga

**Affiliations:** 1 Laboratory of Cardiorespiratory and Metabolic Physiology, Federal University of Uberlândia, Uberlândia 38400-678, Minas Gerais, Brazil; ana.amaral@ufu.br (A.L.A.); jaqueline.batista@imepac.edu.br (J.P.B.); igor.mariano@ufu.br (I.M.M.); ludimila.goncalves@ufu.br (L.F.G.); juliabuiatte@ufu.br (J.B.T.); 2 Laboratory of Biochemistry and Molecular Biology, Institute of Biotechnology, Federal University of Uberlândia, Uberlândia 38400-902, Minas Gerais, Brazil; adrielevieira@ufu.br (A.V.d.S.); douglas.caixeta@ufu.br (D.C.C.); renataroland@ufu.br (R.R.T.); foued@ufu.br (F.S.E.); 3 Laboratory of Nutrition, Exercise and Health (LaNES), School of Medicine, Federal University of Uberlandia (UFU), Uberlândia 38400-902, Minas Gerais, Brazil; erickdeoliveira@ufu.br

**Keywords:** climacteric, exercise, endogenous antioxidants, Pilates training, oxidative stress balance

## Abstract

Postmenopausal women have a high prevalence of cardiometabolic diseases and that may associate with higher oxidative stress. Exercise can contribute to the treatment of such diseases, but some modalities, such as Mat Pilates, need to be further studied in terms of their physiological responses. Our aim was to investigate the effects of 12 weeks of Mat Pilates on redox status in postmenopausal women with one or multiple comorbidities of cardiometabolic diseases. Forty-four postmenopausal women were divided into two groups: SINGLE, composed of women with one cardiometabolic disease (*n* = 20) and MULT, with multimorbidity (*n* = 24). Mat Pilates training was conducted three times a week for 12 weeks, and each session lasted 50 min. Plasma samples were collected before and after training to analyze the following redox markers: superoxide dismutase, catalase, glutathione peroxidase, total antioxidant capacity due to ferric-reducing antioxidant power (FRAP), reduced glutathione (GSH), uric acid, and carbonyl protein. ANCOVA showed interaction effects in FRAP (*p* = 0.014). Both groups had reduced levels of catalase (*p* = 0.240) and GSH (*p* = 0.309), and increased levels of carbonyl protein (*p* = 0.053) after intervention. In conclusion, the redox status of postmenopausal women shows no changes mediated by Mat Pilates training between SINGLE and MULT, except for greater reductions of FRAP in SINGLE.

## 1. Introduction

Multimorbidity is the coexistence of two or more chronic health complications [1], and may include cardiometabolic multimorbidity disease. People with multimorbidity have a higher risk of premature mortality compared to those without disease [2]. In addition, there is an increased incidence of diseases with aging [2], especially cardiometabolic diseases, which are the leading cause of death, hospitalization, and outpatient care worldwide, including in developing countries such as Brazil [3]. Obesity [4], arterial hypertension [3], and dysfunction lipid [5] and glycemic profiles [6] are the most common chronic health complications. On their own, these conditions already have well-defined treatment guidelines, but when associated with other conditions, their potential interactions are not yet well defined [7]. The emergence of these diseases is directly related to the individual’s redox imbalance, trending towards an increase in pro-oxidants [8]. Reactive oxygen species (ROS) play an important role in the pathogenesis of these diseases [9]. In hypertension, they can generate renin-angiotensin system dysfunction [10,11,12], and in diabetes they cause dysregulation of the end products axis of advanced glycation [9]. Furthermore, ROS act directly in the production of atherosclerotic plaques through the oxidation of low-density lipoproteins (LDL) and increase endothelial dysfunction [8].

Postmenopausal women are predisposed to the development of cardiometabolic diseases, mainly due to the cessation of estrogen production, an increase in the concentration of visceral adipose tissue, inflammation, sympathetic activity, production of vasoconstrictors, renal vascular resistance, and increased oxidative stress [13]. However, physical exercise in postmenopausal women has been shown to be an important therapeutic approach treating for cardiometabolic diseases [14], improving bone mineral density [15] and maintenance/gain of lean mass [16]. Aerobic exercise can improve cardiovascular health in this population [14], reduce body mass index (BMI), and promote upregulation of redox status markers, such as superoxide dismutase (SOD) activity, without altering cortisol/leptin levels [17]. Meanwhile, resistance training is capable of improving glycated hemoglobin, muscle strength, waist circumference, and total and LDL cholesterol, as well as preventing the increase in pro-inflammatory markers [16]. Combining these two types of training can lead to an improvement in arterial stiffness [18,19], blood pressure, endothelin-1, blood nitrite/nitrate, functional capacity, and body composition in postmenopausal women [19]. In addition, other physical exercise practices, such as yoga, may improve the level of adiponectin, serum lipids, and metabolic syndrome risk factors in obese postmenopausal women [20]. However, the Pilates training remains unclear.

Mat Pilates has resistance exercise characteristics (with features similar to bodyweight and functional fitness training), using accessories to increase the exercise load, but without using the classic apparatus of the method [21]. While Pilates does not appear on the Fitness Trends list, related terms are found in the global Top 20, such as resistance training, bodyweight training, and functional fitness training [22]. In addition, it is a method with high adherence in the postmenopausal population [23]. Physical training, in turn, is a strategy for the treatment of multimorbidity, as it generates benefits [1] such as psychosocial effects, increased strength, blood pressure regulation and improved insulin sensitivity [24], and has anti-inflammatory effects [25]. However, while exercise can be safely practiced by patients with multimorbidities, there are still no specific recommendations for exercise therapy for this population [26]. In this sense, the effects of physical training in populations with different types of isolated cardiometabolic diseases are relatively well explored; however, the evidence in populations with multimorbidities, especially in populations with an increased risk of these diseases, such as in women after menopause, are still scarce. Furthermore, there are indications that health interventions in individuals with multimorbidities may have minor or insignificant effects [27], and the effects of different kinds of exercise training on anti-inflammatory and antioxidant systems in patients with multimorbidities remain unclear in the literature. Given this gap, understanding the effects of physical training in postmenopausal women with multimorbidities in relation to those with only one cardiometabolic disease is important in the management of these patients with their idiosyncrasies.

Since exercise training is a strategy for the treatment of multimorbidity [1] and can also generate redox adaptations [28], Mat Pilates therapy could be beneficial. However, its effects on populations with cardiometabolic multimorbidity and increased oxidative stress have been poorly explored. Although the consequences of exercise-induced oxidative stress remain a controversial, a moderate level of ROS production during exercise can promote adaptation through mitochondrial biogenesis, antioxidant enzymes, and stress protein synthesis. On the other hand, high levels of ROS production result in damage to macromolecular structures, such as DNA, proteins, and lipids [29]. Thus, our aim was to investigate whether the effects of 12 weeks of Mat Pilates on redox status differ between postmenopausal women with single or multiple cardiometabolic diseases. We hypothesized that exercise training with Mat Pilates could lead to improvements in the redox status in postmenopausal women, regardless of the number of cardiometabolic diseases. This would be less evident in women with multimorbidity due to the association of these diseases with a high level of ROS production.

## 2. Materials and Methods

### 2.1. Participants

This was a parallel clinical trial. All steps of this study were carried out at the Laboratory of Cardiorespiratory and Metabolic Physiology at the Federal University of Uberlândia between August 2017 and March 2019. Data collection started after August 2017. All participants signed the Consent Form and were allocated to groups for convenience in a non-probabilistic way. The present study followed the ethical principles of the Declaration of Helsinki. In addition, the study was approved by the local Ethics Committee of the Federal University of Uberlândia (68408116.9.0000.5152) and was registered on “clinicaltrials.gov” (NCT03626792). This study was part of a larger research project in which the effects of Mat Pilates exercise training on climacteric symptoms, ambulatory blood pressure responses, lipid, and glucose profile and pro- and anti-inflammatory and antioxidant markers in postmenopausal normotensive and hypertensive women, were investigated.

We included in the study (i) post-menopausal women, (ii) aged between 50 and 70 years, (iii) with a low level of physical activity, (iv) who did not present physical limitations that prevented them from performing physical exercise, (v) without hormone therapy, and (vi) who did not smoke. We excluded those women who missed two consecutive training sessions and/or did not complete 80% of the training schedule; who changed their medication or the dose of their medication during the study; and performed physical exercise in parallel with this intervention.

**Figure 1:**
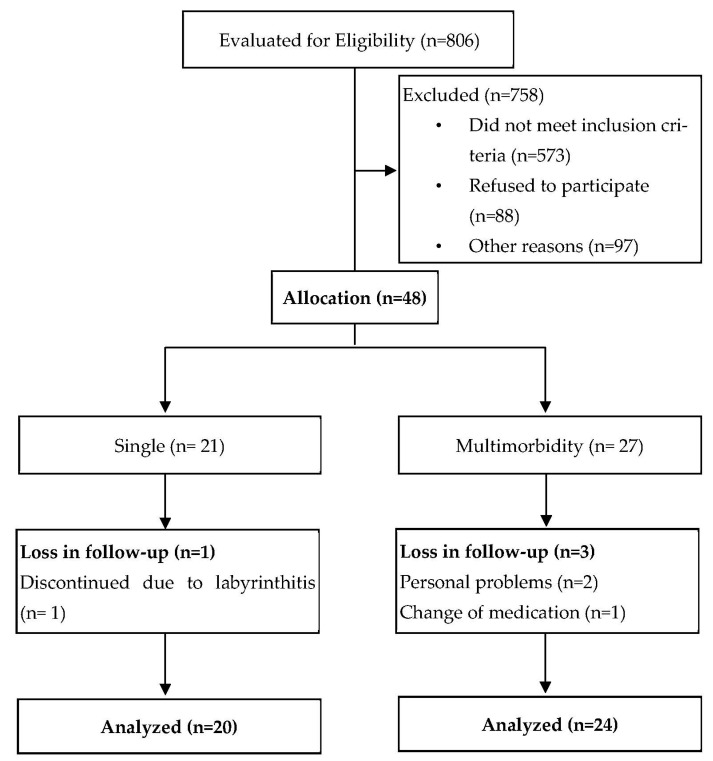
Follow-up flowchart.

Initially, 806 women were registered following wide dissemination of information (TV, radio, and posters), of which 758 were excluded for not meeting the inclusion criteria. Therefore, 48 women were properly allocated to the two groups, and 44 participants completed the entire study (Figure 1).

### 2.2. Procedures

The Pilates exercise program was conducted three times a week for 12 weeks. First, we performed familiarization sessions, explaining the Pilates exercises, its principles, and Borg’s Rating Perceived Exertion 6–20 Scale (RPE; 6 being no exertion and 20 being maximum exertion) [30].

Prior to and 72 h after execution of the training protocol, we performed anthropometric assessments, blood pressure measurements at rest, food recalls, and blood collection. There was no blinding of the participants or evaluators, but the evaluations were always carried out by the same researchers.

After the initial assessment, the volunteers were divided into two groups according to their number of cardiometabolic diseases: SINGLE—women with one cardiometabolic disease; and MULT—women with two or more cardiometabolic diseases (multimorbidity). The criteria for the multimorbidity of cardiometabolic diseases was the coexistence of two or more of the following chronic diseases [31]: blood glucose > 126 mg/dL [6]; obesity (BMI ≥ 30 kg/m^2^ [32]); dyslipidemia (LDL ≥ 160 mg/dL and/or triglycerides ≥ 150 mg/dL and/or total cholesterol ≥ 190 mg/dL and/or high density lipoprotein (HDL) ≤ 50 mg/dL) [5]; hypertension (systolic blood pressure > 139 mmHg and diastolic blood pressure > 89 mmHg) [3]; and/or use of medication to control any of these diseases. Therefore, while participating in the study, volunteers with cardiometabolic diseases were being treated with medication.

### 2.3. Exercise Training Program

Classical Pilates exercises were chosen for the 12-week exercise training program [33]. Two types of sessions (A and B) were performed alternately by all volunteers. These sessions consisted of three warm-up exercises, eight exercises in the main part of the session, and three exercises in the cool down in accordance with our previous study [34] (detailed information about exercises and sessions can be found in Supplementary Table S1). Mat Pilates sessions were conducted in the morning, with a maximum of 10 participants and a duration of 50 min. The first 5 min were dedicated to warming up, and the final 5 min to cooling down. The rest period between exercises was 45 s. The volunteers were instructed to maintain the principles of the method, especially breathing, and to maintain the correct posture during each exercise. All sessions were supervised by certified Pilates exercise specialists. The exercise load progression is presented in Table 1.

### 2.4. Measurement of General Characteristics

Initially, an anamnesis was taken by asking general questions. Stature was measured using a fixed stadiometer (Sanny, São Bernardo do Campo, Brazil) and body mass, and its distribution, were measured by bioimpedance using In Body 230 (Seoul, South Korea). The BMI was calculated using the value of body mass and height, according to the formula: BMI = body mass (kg)/height^2^ (cm). Waist circumference was measured above the umbilical scar using an inelastic flexible measuring tape (Filizola, São Paulo, Brazil) without tissue compression. Blood pressure and resting heart rate were measured before and after training using an OMRON HEM-7113 automatic monitor, properly validated and calibrated. Three measurements of blood pressure and heart rate per day were taken on 3 non-consecutive days, and the means were used for analysis. These assessments always took place in the morning, in a sitting position after resting for 20 min in a silent environment, with restricted conversations and with relaxed arms and legs.

### 2.5. Dietary Intake

Participants answered the 24-h dietary recall administered by trained nutritionists. The volunteers were instructed to remember the foods and beverages they had consumed the day before. To calculate food consumption, three dietary records were collected, on 2 days during the week and one day at the weekend, on nonconsecutive days, and the mean recalls were calculated. Dietary data were evaluated at the beginning of the study and after 12 weeks of intervention. The total intake of energy, protein, fat, carbohydrates, fiber, and micronutrients was quantified using the Dietpro^®^ software (version 5.7i, Agromídia Software^®^, Minas Gerais, Brazil) and the US Department of Agriculture food composition table [35,36] and manufacturers’ nutrition labels.

### 2.6. Blood Collection and Analysis

For the blood samples, 15 mL of blood were collected after an overnight fast, 5 days before and 72 h after the last exercise session. The samples were placed in tubes with EDTA (ethylenediamine tetraacetic acid) or separating gel and centrifuged at 3000 rpm for 15 min, and the supernatant (plasma) was aliquoted. All samples were kept frozen at −80 °C until analysis and biochemical determinations were made in duplicate. The analyses performed were:

- Total protein: performed using the Bradford method and a bovine serum albumin curve [37];
- Enzymatic antioxidants: the activity of superoxide dismutase (SOD), catalase (CAT), and glutathione peroxidase (GPx) was evaluated. SOD activity was evaluated by inhibiting pyrogallol auto-oxidation. Samples were mixed with 50 mmol·L^−1^ Tris-HCl buffer (pH 8.2) containing 1 mmol·L^−1^ EDTA to deactivate metal-dependent enzymes, 80 U·mL^−1^ catalase and 24 mmol·L^−1^ of pyrogallol, and the kinetic assay was monitored for 10 min at 420 nm using an analytical curve constructed with SOD as the standard [38]. CAT activity was based on the decomposition of hydrogen peroxide. The samples were mixed with a 10 mmol·L^−1^ potassium phosphate buffer (pH 7.0 containing 0.2% hydrogen peroxide. The decomposition of hydrogen peroxide was monitored at 240 nm for 10 min [38]. To measure GPx activity, the plasma was incubated with GPx buffer (100 mM potassium phosphate containing 1 mM EDTA, pH 7.7), sodium azide (40 mM), GSH (diluted in 5% metaphosphoric acid), GR (diluted in GPx buffer), NADPH (diluted with 5% sodium bicarbonate) and tert-butyl hydroperoxide (0.5 mM). The reduction in NADPH concentration was evaluated for 10 min in a spectrophotometer at 340 nm [39];
- Total antioxidant capacity due to ferric-reducing antioxidant power (FRAP) was determined by the ability of antioxidants present in blood samples to reduce Fe^3+^ to Fe^2+^ which is chelated by TPTZ (2,4,6-tris(2-pyridyl)-s-triazine) and forms the Fe^2+^ TPTZ complex. This complex was quantified in a spectrophotometer at 593 nm, and antioxidant activity was determined by means of an analytical curve, constructed with trolox as the standard [40];
- Reduced glutathione (GSH): Plasma proteins were precipitated with metaphosphoric acid (MPA) and centrifuged at 7000× *g* for 10 min. The supernatant was collected and mixed with 100 mM sodium phosphate buffer (pH 8.0) containing 5 mM EDTA and 15 μL of ortho-phthaldialdehyde (1 mg/mL in methanol, for HPLC, ≥ 99.9%). The solution was incubated in the dark at room temperature for 15 min. Fluorescence was read at 350 nm (excitation) and 420 nm (emission). Sample GSH concentrations were calculated using a standard curve of GSH (0.001–0.1 mM) [40];
- Uric acid: analysis performed by the automated uricase-PAP method;
- Carbonylated protein: Protein carbonyls were identified by 2,4-dinitrophenylhydrazine (DNPH). Samples were incubated with 10 mM DNPH (diluted in 2.5 N HCl) for 1 h and then precipitated with 20% trichloroacetic acid (TCA). After centrifugation (9000× *g* for 5 min), the pellet was washed with ethanol-ethyl acetate and resuspended in guanidine hydrochloride 6 mol.L^−1^ (diluted in 2.5 N HCl). A blank was performed with the sample incubated with 2.5 N HCl (without DNPH) and followed the same procedures, in order to reduce the contribution of the color to the absorbance. Absorbance values were recorded at 370 nm (Molecular Devices, Menlo Park, CA, 213 USA) [38].

### 2.7. Statistical Analysis

We were unable to find any studies that assessed postmenopausal women with enough similarities multimorbidities undergoing exercise training to justify their use for a priori sample calculation, and, therefore, we chose to present the power analysis values found a posteriori by G*power 3.1.9.7 in the results section (α = 0.05; 2 groups, *n* = 47, effect sizes based on groups delta). The results are presented as mean ± standard deviation. To verify the normality of the results, we applied the Shapiro–Wilk test, and for the homogeneity of variance, we used the Levene test. Baseline characteristics of the groups were compared using an unpaired t-test. Variables that did not meet the assumptions of normal distribution and homogeneity of variance were evaluated using the Mann–Whitney test. Analysis of covariance (ANCOVA) was used to compare the redox markers and food consumption between groups. This comparison was adjusted according to baseline values due the high individual variance of redox markers values. Data are presented in both pre- and post-adjusted. Variation between pre- and post-training and confidence intervals were calculated from the unadjusted post-training data. Effect sizes were calculated using Cohen’s d, from the variation between pre-and post-training groups. All analyses were performed using SPSS software version 21.0. The level of significance adopted was *p* < 0.05.

## 3. Results

Of the 48 volunteers who started the study, a total of 44 volunteers completed it, 20 participants in SINGLE and 24 in MULT. One volunteer from the SINGLE group discontinued the study due to labyrinthitis, and 3 from the MULT group discontinued due to personal problems or medication modifications. None of the participants had training-related injuries, and they had performed at least 33 of the 36 sessions. The general characteristics of the sample are shown in Table 2. We found that MULT had lower triglycerides (*p* = 0.050) and a higher BMI (*p* = 0.003) and waist circumference [*p* = 0.0002; MULT had a substantially increased cardiovascular risk (≥ 88 cm) and SINGLE had a normal to increased risk (≥ 80 cm) [41]], and there were no obese women in SINGLE. At rest, the values of blood pressure, blood glucose, and lipid profile were not different between groups. The mean value and percentage of drugs used that control cardiometabolic diseases are shown in Table 2. We analyzed dietary recall data from SINGLE (*n* = 13) and MULT (*n* = 20) and found no significant differences in baseline or training effects for macronutrients, saturated and unsaturated lipids, cholesterol, fiber, zinc, vitamin A, and vitamin C (Supplementary Table S2). Data are presented in pre-training and post-training adjusted by ANCOVA for the baseline value.

Table 3 presents the redox data measured in plasma and the data are presented as pre-training and post-training adjusted by ANCOVA for the baseline value. This analysis showed interaction effects only in FRAP (Δ% = 12 in SINGLE, and 8 in MULT; ES = 0.908; *p* = 0.014). Additionally, there was no significant difference at baseline between groups in any of the analyzed variables (SOD, *p* = 0.28; CAT, *p* = 0.46; GPx, *p* = 0.90; FRAP *p* = 0.90; GSH *p* = 0.44; carbonylated protein *p* = 0.88). Although the ANCOVA did not show isolated exercise training effects, the results can be explored using 95% confidence intervals. These showed a reduction in both groups in catalase [∆ = −577.34 (−702.27 to −452.41) for SINGLE; ∆ = −540.87 (−733.64 to −348.1) for MULT], and in GSH [∆ = −1.93 (−2.34 to −1.52) for SINGLE; ∆ = −2.75 (−3.32 to −2.18) for MULT] and an increase in carbonylated protein [∆ = 1.31 (0.95 to 1.67) for SINGLE; ∆ = 1.68 (1.27 to 2.09) for MULTI]. Table 4 shows the achieved power analysis for the redox data.

## 4. Discussion

Our study investigated whether 12 weeks of Mat Pilates exercise training could affect the redox status in postmenopausal women with single or multiple cardiometabolic diseases. Our main result shows no significant baseline group differences in the redox status. We also found no significant changes in most of the markers of redox status after the exercise training program in either group. Only FRAP differed significantly after training with Mat Pilates, with confidence intervals showing a greater reduction in the SINGLE group. Moreover, the baseline values of blood pressure, blood glucose, and lipid profile did not differ between groups due to the use of drugs to control cardiometabolic diseases; however, there were differences in for BMI and waist circumference, which were higher in women with multimorbidity as expected.

In previous studies, postmenopausal women [42] and hypertensive individuals [43] had lower FRAP values, demonstrating the presence of oxidative damage. The ferric-reducing ability of plasma, FRAP, has been used as an indirect measurement to determine the total antioxidant activity, and some studies have shown high values of this marker in patients with one or more morbidity such as hypertension, obesity, diabetes, and metabolic syndrome [44]. However, the correlation between FRAP and other enzymatic antioxidant markers such as GSH and uric acid measured in human blood remains controversial [44,45,46,47]. The lack of correlation among redox biomarkers could be due to the inactivation of these enzymes under conditions of high oxidative stress [45,46].

In the present study, the SINGLE group showed greater reductions in FRAP values when compared to MULT, which is consistent with Smith et al. [27], who found that individuals with multimorbidities may have experience only minor or negligible effects of physical training. In postmenopausal women, the reduction in 17β-estradiol directly impacts aspects of mitochondrial function, including the production of ROS and ATP, and the membrane potential [48]. Considering that cardiometabolic diseases and the climacteric are associated with oxidative stress, these women probably started the exercise protocol under higher oxidative stress [8,42,49,50,51,52]. There is a lack of research investigating the effects of the Pilates method on redox status; therefore, given that this method has some characteristics similar to resistance and/or isometric training method, we can compare our results with studies using those types of exercise. However, we know that even though they share similar characteristics, it would be better to compare our results with other studies using the Pilates method. Although training with Mat Pilates is an interesting proposal for adherence for this group moderate-intensity aerobic exercise has better effects on oxidative stress in hypertensive postmenopausal women [14] and also in type 2 diabetic individuals [53].

A study with elderly women undergoing 12 weeks of resistance training showed that there was a reduction in uric acid and an increase in catalase and SOD [54], contradicting our findings. In older adults with and without metabolic syndrome undergoing 6 months of aerobic training, there were no significant differences in most of the oxidative stress variables, including protein carbonylation, antioxidant enzymes, and uric acid [55]. In addition, women with metabolic syndrome had significantly higher FRAP levels compared to women without metabolic syndrome [55], showing an increase in antioxidant capacity probably generated by increased production of pro-oxidants. Therefore, although physical training is considered the cornerstone of non-pharmacological therapy for postmenopausal hypertension [14], the oxidative profile responses in populations of postmenopausal women and those associated with cardiometabolic disease still need to be further elucidated.

Carbonyl protein can be used as a marker of protein damage, given that postmenopausal women showed an increase in its expression when compared to pre-menopausal women [13]. In patients with chronic obstructive pulmonary disease, lower values of antioxidant enzymes and higher values of protein carbonylation were found after training, probably due to disturbances in the mitochondrial respiratory chain [56].

We found no difference in the baseline of the redox status between variables between the SINGLE versus MULT groups. This may be due to the following possibilities: unlike SINGLE, half of the MULT sample had a BMI classifying them as obese 1 [41], in addition to a higher waist circumference. In this sense, MULT presents a substantially increased cardiovascular risk while SINGLE had a normal to increased risk [41]. However, the lipid profile data did not differ significantly, and the total and LDL cholesterol values for both groups were above the recommended level (<190 mg/dL for total cholesterol; <130 for LDL) [5], which could be problematic because oxidized LDL stimulates inflammation and the accumulation of ROS generates damage [8]. Moreover, the fact that MULT were well treated by drugs may have led to similar responses to the SINGLE group.

When we analyzed the preexisting cardiometabolic diseases in MULT, we found that 46% of the volunteers were obese and 83% hypertensive. Obesity is related to oxidative stress without a well-defined causal direction. Oxidative stress can stimulate white adipose tissue deposition and increase preadipocyte proliferation, adipocyte differentiation, and mature adipocyte size and obesity can elevate chronic inflammation with increased oxidative stress [57]. Increased oxidative stress and redox imbalance damage cell structures, generate underproduction of antioxidant mechanisms, and modify mitochondrial activity, leading to the development of obesity-related complications [49,57]. This pro-oxidant state could explain why we did not find changes in plasma oxidative markers after 12 weeks of Mat Pilates training. Hypertension, on the other hand, can intensify oxidative stress through increased lipid peroxidation, endothelial dysfunction, and inflammation caused by the sustained increase in blood pressure values [50]. However, the present study found similar results in both groups, since both groups had the same types of morbidities (differentiated by the amount), and despite having isolated effects on the redox status, an addition effect of these results has not yet been demonstrated.

Lastly, despite the presence of hypertension, all study participants were taking drugs to control blood pressure. However, the use of antihypertensive drugs could also explain the absence of differences in redox biomarkers found between the SINGLE and MULT groups. The antihypertensive drugs used by volunteers act mainly on the renin-angiotensin system. Both angiotensin converting enzyme inhibitors and angiotensin receptor antagonists inhibit vascular remodeling and reduce ROS [58] through the reduction of NADPH oxidase and upregulation of Cu/ZnSOD [11,12]. These drugs can also improve endothelial function [58] and appear to be mediated by decreased lipoxygenase enzyme expression [59]. In addition, by decreasing Angiotensin II, angiotensin-converting enzyme inhibition, limits the stimulation of vascular NAD(P)H oxidase, preventing the increase in superoxide flow associated with activation of the renin-angiotensin system. Finally, angiotensin-converting enzyme inhibitors also limit the formation of hydrogen peroxide [59,60] and lipid peroxidation by the reduction of peroxynitrite formation [60]. As for thiazide diuretics, there is scant evidence of effects on oxidative stress [61,62]; however, it may be that they act on the antioxidant protection expressed by higher levels of FRAP [62].

For dietary intake, there are indications that the high intake of macronutrients, especially saturated fats and refined carbohydrates consumed in excess, can promote oxidative stress and, consequently, contribute to inflammation [63,64]. On the other hand, fiber, vitamin C, and some unsaturated lipids can act as antioxidants, contributing to the reduction of oxidative stress [64]. Although consumption of these nutrients is related to the increase in cardiometabolic diseases and oxidative stress [64], we did not find changes in intake during the training protocol. Therefore, the results found in this study are probably not related to food consumption by the sample.

Regarding the potential clinical application of this study, Mat Pilates is a type of exercise with high adherence in the postmenopausal population [23]. Furthermore, despite not having found significant differences in the redox parameters, the Pilates method has relevance for other clinical parameters, such as the improvement of sexual function and the quality of sexual life of climacteric women [65], and physical and motor benefits in the elderly [66]. Therefore, understanding the physiological and clinical responses of the Pilates method, associated or not with cardiometabolic multimorbidity, is important when thinking about the best training approach for the climacteric population, given the scarcity and/or low methodological quality of previous studies [66].

This study had some limitations. Some redox markers presented a low or moderate power in the statistical analysis, and this could be the reason for the lack of results in some variables. Having a control group without training could help us understand the effects of Mat Pilates, not just differences in responses between populations; however, we did not have this group, as our aim was to compare the exercise-mediated responses in women with single or multiple cardiovascular disease. In addition, the volunteers were medicated, which may make it difficult to generalize these results to populations with non-existent or less effective treatments. Another limitation we had was that not all volunteers answered the dietary recall, and dietary patterns were not controlled for all participants. In addition, there was an imbalance between the number of pro-oxidant and antioxidant markers analyzed in the present study, which limits our interpretation of the results in relation to the global redox status, but prioritizes the expected antioxidant results of physical training. In this sense, there are other markers that we did not analyze that could increase our understanding of the redox status of these women, such as the metabolism of lipid peroxidation and other non-enzymatic and exogenous antioxidants. Therefore, future studies should include participants with more severe conditions of cardiometabolic multimorbidity and a protocol that uses a control group that does not practice Mat Pilates.

## 5. Conclusions

We conclude that there were no changes in redox status mediated by 12 weeks of Mat Pilates exercise training between SINGLE and MULT, except that FRAP in SINGLE was lower. This study points to new perspectives to investigate in patients with multimorbidities the impacts of this physical training program on changes in the systemic redox state.

## Figures and Tables

**Table 1:** Exercise load progression.

Weeks of Training	Exercise Load Progression
1–3rd	10 repetitions, body weight
4–6th	12 repetitions, body weight
7–9th	12 repetitions, shin guards and free weight
10–12th	15 repetitions, shin guards and free weight

**Table 2:** Baseline general characteristics, drugs, and comorbidities.

	SINGLE	MULT	TOTAL
	*n* = 20	*n* = 24	*n* = 44
Characteristics			
Age (years)	57 ± 4	58 ± 6	58 ± 5
Postmenopausal time (years)	10 ± 6	9 ± 7	9 ± 7
Waist circumference (cm)	79 ± 7	96 ± 9 *	84 ± 8
BMI (kg/m^2^)	25 ± 3	29 ± 4 *	27 ± 4
Obesity (*n* (%))	0 (0)	11 (46)	11 (25)
Rest SBP (mmHg)	121 ± 8	119 ± 10	120 ± 9
Rest DBP (mmHg)	77 ± 8	76 ± 8	77 ± 8
Hypertension (*n* (%))	3 (15)	20 (83)	23 (52)
Fasting glucose (mg/dL)	97 ± 10	93 ± 8	95 ± 9
Diabetes Mellitus (*n* (%))	0 (0)	0 (0)	0 (0)
Total cholesterol (mg/dL)	231 ± 40	218 ± 35	224 ± 37
HDL (mg/dL)	60 ± 11	61 ± 11	61 ± 11
LDL (mg/dL)	142 ± 39	135 ± 33	138 ± 36
Triglycerides (mg/dL)	162 ± 89	113 ± 44 *	135 ± 72
Dyslipidemia (*n* (%))	17 (85)	22 (92)	39 (89)
Drugs (*n* (%))			
Statins	1 (5)	1 (4)	2 (5)
ARB	1 (5)	6 (5)	7 (16)
ARB + Diuretic	2 (10)	7 (29)	9 (20)
Diuretic	0 (0)	2 (9)	2 (5)
ACEi	0 (0)	4 (17)	4 (9)
ACEi + Diuretic	0 (0)	1 (4)	1 (2)
Levothyroxine	3 (15)	3 (13)	6 (14)
Number of comorbidities (*n* (%))			
One	20 (100)	0 (0)	20 (45)
Two	0 (0)	20 (83)	20 (43)
Three	0 (0)	4 (17)	4 (9)

Data are presented as mean ± standard deviation or *n* (%). SINGLE: women with 1 disease; MULT: women with ≥ 2 comorbidities; BMI: body mass index; SBP: systolic blood pressure; DBP: diastolic blood pressure; HDL: high density lipoprotein; LDL: low density lipoprotein; ARB: angiotensin receptor blocker; ACEi: Angiotensin Converting Enzyme Inhibitor. * *p* < 0.05, significant difference between groups. Obesity was considered with BMI ≥ 30 (kg/m^2^). Diabetes Mellitus was considered with fasting glucose > 126 mg/dL.

**Table 3:** Plasma redox status in SINGLE (*n* = 20) and MULT (*n* = 24) before and after 12 weeks of Mat Pilates training.

	PRE	POST Adjusted	Mean Difference	ANCOVA *p*
	Mean ± SD	Mean ± SD	(95%CI)	
SOD (U/mL)				
SINGLE	0.93 ± 0.04	0.82 ± 0.16	−0.11 (−0.16 to −0.06)	0.972
MULT	0.88 ± 0.17	0.82 ± 1.48	−0.06 (−0.48 to 0.36)	
Catalase (U/μg prot)				
SINGLE	1674.48 ± 186.16	1097.14 ± 357.57	−577.34 (−702.27 to −452.41)	0.240
MULT	1508.66 ± 598.25	967.79 ± 326.21	−540.87 (−733.64 to −348.1)	
GPx (U/mg prot)				
SINGLE	1.20 ± 0.07	1.11 ± 0.41	−0.09 (−0.22 to 0.04)	0.102
MULT	1.23 ± 0.29	0.90 ± 0.38	−0.33 (−0.47 to −0.19)	
FRAP (nmol Trolox)				
SINGLE	178.89 ± 5.32	157.12 ± 29.70	−21.77 (−31.12 to −12.42)	0.014
MULT	195.58 ± 83.75	180.38 ± 27.09	−15.2 (−40.1 to 9.7)	
GSH (nmol/mg prot)				
SINGLE	5.96 ± 0.45	4.03 ± 1.24	−1.93 (−2.34 to −1.52)	0.309
MULT	6.39 ± 1.68	3.64 ± 1.13	−2.75 (−3.32 to −2.18)	
Uric acid (mg/dL)				
SINGLE	4.05 ± 0.25	3.81 ± 0.76	−0.24 (−0.49 to 0.01)	0.418
MULT	4.22 ± 1.14	4.10 ± 0.70	−0.12 (−0.5 to 0.26)	
Carbonylated protein (nmol/mg of protein)				
SINGLE	3.05 ± 0.16	4.36 ± 1.16	1.31 (0.95 to 1.67)	0.053
MULT	3.14 ± 0.99	4.82 ± 1.06	1.68 (1.27 to 2.09)	

Data are presented as mean ± standard deviation. SINGLE: women with 1 disease; MULT: women with ≥ 2 comorbidities. Data are presented in pre- and post-adjusted. Mean difference from pre- to post-intervention values were calculated from the unadjusted post. SOD: superoxide dismutase; CAT: catalase; GPx: glutathione peroxidase; FRAP: Total antioxidant capacity due to ferric-reducing antioxidant power; GSH: reduced glutathione.

**Table 4:** Statistical power of analysis.

	Power
SOD (U/mL)	0.050
Catalase (U/μg prot)	0.999
GPx (U/mg prot)	0.167
FRAP (nmol Trolox)	0.908
GSH (nmol/mg prot)	0.999
Uric acid (mg/dL)	0.277
Carbonylated Protein (nmol/mg of prot)	0.999

SOD: superoxide dismutase; CAT: catalase; GPx: glutathione peroxidase; FRAP: Total antioxidant capacity due to ferric-reducing antioxidant power; GSH: reduced glutathione.

## Data Availability

The general characteristics and redox data used to support the findings of this study are available from the corresponding author upon request.

## Supplementary Materials

The following supporting information can be downloaded at: https://www.mdpi.com/article/10.3390/antiox11081445/s1. Table S1: Mat Pilates exercise program; Table S2: Analysis of food consumption pattern in SINGLE (*n* = 13) and MULT (*n* = 20) before and after 12 weeks of Mat Pilates training.

## References

[1] Bricca A., Harris L.K., Jäger M., Smith S.M., Juhl C.B., Skou S.T. (2020). Benefits and Harms of Exercise Therapy in People with Multimorbidity: A Systematic Review and Meta-Analysis of Randomised Controlled Trials. Ageing Res. Rev..

[2] Di Angelantonio E., Kaptoge S., Wormser D., Willeit P., Butterworth A.S., Bansal N., O’Keeffe L.M., Gao P., Wood A.M., Burgess S. (2015). Association of Cardiometabolic Multimorbidity with Mortality. JAMA.

[3] Barroso W., Rodrigues C., Bortolotto L., Gomes M., Brandão A., Feitosa A. (2020). Diretrizes Diretrizes Brasileiras de Hipertensão Arterial—2020. Arq. Bras. Cardiol..

[4] Mancini M.C., Associação Brasileira para o estudo da obesidade e da síndrome metabólica (ABESO) (2016). Diretrizes Brasileiras de Obesidade 2016. VI Diretrizes Bras. Obesidade.

[5] Faludi A., Izar M., Saraiva J., Chacra A., Bianco H., Afiune Neto A., Bertolami A., Pereira A., Lottenberg A., Sposito A. (2017). Atualização Da Diretriz Brasileira de Dislipidemias e Prevenção Da Aterosclerose. Arq. Bras. Cardiol..

[6] Lyra R., Oliveira M., Lins D., Cavalcanti N., Gross J.L., Maia F.F.R., Araújo L.R., Yafi M., Guimarães F.P.D.M., Takayanagui A.M.M., Bertoluci M. (2020). Sociedade Brasileira de Diabetes.

[7] Ho I.S.-S., Azcoaga-Lorenzo A., Akbari A., Black C., Davies J., Hodgins P., Khunti K., Kadam U., Lyons R.A., McCowan C. (2021). Examining Variation in the Measurement of Multimorbidity in Research: A Systematic Review of 566 Studies. Lancet Public Health.

[8] Kattoor A.J., Pothineni N.V.K., Palagiri D., Mehta J.L. (2017). Oxidative Stress in Atherosclerosis. Curr. Atheroscler. Rep..

[9] Santilli F., D’Ardes D., Davì G. (2015). Oxidative Stress in Chronic Vascular Disease: From Prediction to Prevention. Vascul. Pharmacol..

[10] Petrie J.R., Guzik T.J., Touyz R.M. (2018). Diabetes, Hypertension and Cardiovascular Disease: Clinical Insights and Vascular Mechanisms. Can. J. Cardiol..

[11] Tanaka M., Umemoto S., Kawahara S., Kubo M., Itoh S., Umeji K., Matsuzaki M. (2005). Angiotensin II Type 1 Receptor Antagonist and Angiotensin-Converting Enzyme Inhibitor Altered the Activation of Cu/Zn-Containing Superoxide Dismutase in the Heart of Stroke-Prone Spontaneously Hypertensive Rats. Hypertens. Res..

[12] Landmesser U., Drexler H. (2003). Oxidative Stress, the Renin-Angiotensin System and Atherosclerosis. Eur. Heart J. Suppl..

[13] Montoya-Estrada A., Velázquez-Yescas K.G., Veruete-Bedolla D.B., Ruiz-Herrera J.D., Villarreal-Barranca A., Romo-Yañez J., Ortiz-Luna G.F., Arellano-Eguiluz A., Solis-Paredes M., Flores-Pliego A. (2020). Parameters of Oxidative Stress in Reproductive and Postmenopausal Mexican Women. Int. J. Environ. Res. Public Health.

[14] Lin Y.-Y., Lee S.-D. (2018). Cardiovascular Benefits of Exercise Training in Postmenopausal Hypertension. Int. J. Mol. Sci..

[15] Daly R.M., Dalla Via J., Duckham R.L., Fraser S.F., Helge E.W. (2018). Exercise for the Prevention of Osteoporosis in Postmenopausal Women: An Evidence-Based Guide to the Optimal Prescription. Braz. J. Phys. Ther..

[16] Nunes P.R.P., Barcelos L.C., Oliveira A.A., Furlanetto Júnior R., Martins F.M., Orsatti C.L., Resende E.A.M.R., Orsatti F.L. (2016). Effect of Resistance Training on Muscular Strength and Indicators of Abdominal Adiposity, Metabolic Risk and Inflammation in Postmenopausal Women: Controlled and Randomized Clinical Trial of Efficacy of Training Volume. Age.

[17] Jarrete A.P., Novais I.P., Nunes H.A., Puga G.M., Delbin M.A., Zanesco A. (2014). Influence of Aerobic Exercise Training on Cardiovascular and Endocrine-Inflammatory Biomarkers in Hypertensive Postmenopausal Women. J. Clin. Transl. Endocrinol..

[18] Manojlović M., Protić-Gava B., Maksimović N., Šćepanović T., Poček S., Roklicer R., Drid P. (2021). Effects of Combined Resistance and Aerobic Training on Arterial Stiffness in Postmenopausal Women: A Systematic Review. Int. J. Environ. Res. Public Health.

[19] Son W.-M., Sung K.-D., Cho J.-M., Park S.-Y. (2017). Combined Exercise Reduces Arterial Stiffness, Blood Pressure and Blood Markers for Cardiovascular Risk in Postmenopausal Women with Hypertension. Menopause.

[20] Lee J.-A., Kim J.-W., Kim D.-Y. (2012). Effects of Yoga Exercise on Serum Adiponectin and Metabolic Syndrome Factors in Obese Postmenopausal Women. Menopause.

[21] Batista J.P., Mariano I.M., Amaral A.L., Matias L.A.S., De Souza T.C.F., Resende A.P.M., Puga G.M. (2021). Acute Effects of Mat Pilates on Ambulatory Blood Pressure Variability in Post Menopause Women. Rev. Bras. Fisiol. Exerc..

[22] Kercher V.M., Kercher K., Bennion T., Yates B.A., Feito Y., Alexander C., Amaral P.C., Soares W., Li Y.-M., Han J. (2021). Fitness Trends From Around the Globe. ACSM’s Health Fit. J..

[23] Fourie M., Gildenhuys G., Shaw I., Shaw B., Toriola A., Goon D. (2013). Effects of a Mat Pilates Programme on Body Composition in Elderly Women. West Indian Med. J..

[24] Pedersen B.K., Saltin B. (2015). Exercise as Medicine—Evidence for Prescribing Exercise as Therapy in 26 Different Chronic Diseases. Scand. J. Med. Sci. Sports.

[25] Gleeson M., Bishop N.C., Stensel D.J., Lindley M.R., Mastana S.S., Nimmo M.A. (2011). The Anti-Inflammatory Effects of Exercise: Mechanisms and Implications for the Prevention and Treatment of Disease. Nat. Rev. Immunol..

[26] Muth C., Blom J.W., Smith S.M., Johnell K., Gonzalez-Gonzalez A.I., Nguyen T.S., Brueckle M.-S., Cesari M., Tinetti M.E., Valderas J.M. (2019). Evidence Supporting the Best Clinical Management of Patients with Multimorbidity and Polypharmacy: A Systematic Guideline Review and Expert Consensus. J. Intern. Med..

[27] Smith S.M., Wallace E., O’Dowd T., Fortin M. (2016). Interventions for Improving Outcomes in Patients with Multimorbidity in Primary Care and Community Settings. Cochrane Database Syst. Rev..

[28] Ismaeel A., Holmes M., Papoutsi E., Panton L., Koutakis P. (2019). Resistance Training, Antioxidant Status and Antioxidant Supplementation. Int. J. Sport Nutr. Exerc. Metab..

[29] Powers S.K., Deminice R., Ozdemir M., Yoshihara T., Bomkamp M.P., Hyatt H. (2020). Exercise-Induced Oxidative Stress: Friend or Foe?. J. Sport Health Sci..

[30] Borg G.A. (1982). Psychophysical Bases of Perceived Exertion. Med. Sci. Sports Exerc..

[31] Diederichs C., Berger K., Bartels D.B. (2011). The Measurement of Multiple Chronic Diseases—A Systematic Review on Existing Multimorbidity Indices. J. Gerontol. Ser. A Biol. Sci. Med. Sci..

[32] WHO (2017). Global Nutrition Monitoring Framework.Operational Guidance for Tracking Progress in Meeting Targets for 2025.

[33] Pilates J.H., Miller W.J., Robbins J. (1998). Return to Life Trough Contrology.

[34] Batista J.P., Tavares J.B., Gonçalves L.F., de Souza T.C.F., Mariano I.M., Amaral A.L., Rodrigues M.d.L., Matias L.A.S., Magalhães Resende A.P., Puga G.M. (2022). Mat Pilates Training Reduces Blood Pressure in Both Well-Controlled Hypertensive and Normotensive Postmenopausal Women: A Controlled Clinical Trial Study. Clin. Exp. Hypertens..

[35] United States Department of Agriculture (2006). Food Composition Databases.

[36] United States Department of Agriculture (2007). Food Composition Databases.

[37] Bradford M.M. (1976). A Rapid and Sensitive Method for the Quantitation of Microgram Quantities of Protein Utilizing the Principle of Protein-Dye Binding. Anal. Biochem..

[38] Justino A.B., Pereira M.N., Peixoto L.G., Vilela D.D., Caixeta D.C., de Souza A.V., Teixeira R.R., Silva H.C.G., de Moura F.B.R., Moraes I.B. (2017). Hepatoprotective Properties of a Polyphenol-Enriched Fraction from Annona Crassiflora Mart.Fruit Peel against Diabetes-Induced Oxidative and Nitrosative Stress. J. Agric. Food Chem..

[39] Caixeta D.C., Teixeira R.R., Peixoto L.G., Machado H.L., Baptista N.B., de Souza A.V., Vilela D.D., Franci C.R., Salmen Espindola F. (2018). Adaptogenic Potential of Royal Jelly in Liver of Rats Exposed to Chronic Stress. PLoS ONE.

[40] Teixeira R.R., de Souza A.V., Peixoto L.G., Machado H.L., Caixeta D.C., Vilela D.D., Baptista N.B., Franci C.R., Espindola F.S. (2017). Royal Jelly Decreases Corticosterone Levels and Improves the Brain Antioxidant System in Restraint and Cold Stressed Rats. Neurosci. Lett..

[41] World Health Organization (2000). Obesity: Preventing and Managing the Global Epidemic.

[42] Zovari F., Parsian H., Bijani A., Moslemnezhad A., Shirzad A. (2020). Evaluation of Salivary and Serum Total Antioxidant Capacity and Lipid Peroxidation in Postmenopausal Women. Int. J. Dent..

[43] Verma M.K., Jaiswal A., Sharma P., Kumar P., Narayan Singh A. (2019). Oxidative Stress and Biomarker of TNF-α, MDA and FRAP in Hypertension. J. Med. Life.

[44] Gawlik K., Naskalski J.W., Fedak D., Pawlica-Gosiewska D., Grudzień U., Dumnicka P., Małecki M.T., Solnica B. (2016). Markers of Antioxidant Defense in Patients with Type 2 Diabetes. Oxid. Med. Cell. Longev..

[45] Serafini M., Del Rio D. (2004). Understanding the Association between Dietary Antioxidants, Redox Status and Disease: Is the Total Antioxidant Capacity the Right Tool?. Redox Rep..

[46] Rahbani-Nobar M.E., Rahimi-Pour A., Rahbani-Nobar M., Adi-Beig F., Mirhashemi S.M. (1999). Total Antioxidant Capacity, Superoxide Dismutase and Glutathione Peroxidase in Diabetic Patients. Med. J. Islam. Acad. Sci..

[47] Benzie I.F., Strain J.J. (1996). The Ferric Reducing Ability of Plasma (FRAP) as a Measure of “Antioxidant Power”: The FRAP Assay. Anal. Biochem..

[48] Torres M.J., Kew K.A., Ryan T.E., Pennington E.R., Lin C.-T., Buddo K.A., Fix A.M., Smith C.A., Gilliam L.A., Karvinen S. (2018). 17β-Estradiol Directly Lowers Mitochondrial Membrane Microviscosity and Improves Bioenergetic Function in Skeletal Muscle. Cell Metab..

[49] Rani V., Deep G., Singh R.K., Palle K., Yadav U.C.S. (2016). Oxidative Stress and Metabolic Disorders: Pathogenesis and Therapeutic Strategies. Life Sci..

[50] Bielli A., Scioli M.G., Mazzaglia D., Doldo E., Orlandi A. (2015). Antioxidants and Vascular Health. Life Sci..

[51] Signorelli S.S., Neri S., Sciacchitano S., Di Pino L., Costa M.P., Marchese G., Celotta G., Cassibba N., Pennisi G., Caschetto S. (2006). Behaviour of Some Indicators of Oxidative Stress in Postmenopausal and Fertile Women. Maturitas.

[52] Alikhani S., Sheikholeslami-Vatani D. (2019). Oxidative Stress and Anti-oxidant Responses to Regular Resistance Training in Young and Older Adult Women. Geriatr. Gerontol. Int..

[53] De Oliveira V.N., Bessa A., Jorge M.L.M.P., Oliveira R.J.D.S., de Mello M.T., De Agostini G.G., Jorge P.T., Espindola F.S. (2012). The Effect of Different Training Programs on Antioxidant Status, Oxidative Stress and Metabolic Control in Type 2 Diabetes. Appl. Physiol. Nutr. Metab..

[54] Nabuco H.C.G., Tomeleri C.M., Fernandes R.R., Sugihara Junior P., Venturini D., Barbosa D.S., Deminice R., Sardinha L.B., Cyrino E.S. (2019). Effects of Pre- or Post-exercise Whey Protein Supplementation on Oxidative Stress and Antioxidant Enzymes in Older Women. Scand. J. Med. Sci. Sports.

[55] Rytz C.L., Pialoux V., Mura M., Martin A., Hogan D.B., Hill M.D., Poulin M.J. (2020). Impact of Aerobic Exercise, Sex and Metabolic Syndrome on Markers of Oxidative Stress: Results from the Brain in Motion Study. J. Appl. Physiol..

[56] Pinho R.A., Chiesa D., Mezzomo K.M., Andrades M.E., Bonatto F., Gelain D., Dal Pizzol F., Knorst M.M., Moreira J.C.F. (2007). Oxidative Stress in Chronic Obstructive Pulmonary Disease Patients Submitted to a Rehabilitation Program. Respir. Med..

[57] Marseglia L., Manti S., D’Angelo G., Nicotera A., Parisi E., Di Rosa G., Gitto E., Arrigo T. (2014). Oxidative Stress in Obesity: A Critical Component in Human Diseases. Int. J. Mol. Sci..

[58] Silva I.V.G., de Figueiredo R.C., Rios D.R.A. (2019). Effect of Different Classes of Antihypertensive Drugs on Endothelial Function and Inflammation. Int. J. Mol. Sci..

[59] Hanif K., Bid H.K., Konwar R. (2010). Reinventing the ACE Inhibitors: Some Old and New Implications of ACE Inhibition. Hypertens. Res..

[60] Münzel T., Keaney J. (2001). Are ACE Inhibitors a “Magic Bullet” against Oxidative Stress?. Circulation.

[61] Zhou M.S., Schulman I.H., Jaimes E.A., Raij L. (2008). Thiazide Diuretics, Endothelial Function and Vascular Oxidative Stress. J. Hypertens..

[62] Savav T., Cecen F., Albayrak E.S. (2017). The Effects of Ultrafiltration and Diuretic Therapies on Oxidative Stress Markers in Patients with Cardio-Renal Syndrome. Minerva Urol. Nefrol..

[63] Fernández-Sánchez A., Madrigal-Santillán E., Bautista M., Esquivel-Soto J., Morales-González Á., Esquivel-Chirino C., Durante-Montiel I., Sánchez-Rivera G., Valadez-Vega C., Morales-González J.A. (2011). Inflammation, Oxidative Stress and Obesity. Int. J. Mol. Sci..

[64] Tan B.L., Norhaizan M.E., Liew W.-P.-P. (2018). Nutrients and Oxidative Stress: Friend or Foe?. Oxid. Med. Cell. Longev..

[65] Carcelén-Fraile M.D.C., Aibar-Almazán A., Martínez-Amat A., Cruz-Díaz D., Díaz-Mohedo E., Redecillas-Peiró M.T., Hita-Contreras F. (2020). Effects of Physical Exercise on Sexual Function and Quality of Sexual Life Related to Menopausal Symptoms in Peri- and Postmenopausal Women: A Systematic Review. Int. J. Environ. Res. Public Health.

[66] Engers P.B., Rombaldi A.J., Portella E.G., da Silva M.C. (2016). The Effects of the Pilates Method in the Elderly: A Systematic Review. Rev. Bras. Reumatol..

