# Supplementary material for: Redox Status of Postmenopausal Women with Single or Multiple Cardiometabolic Diseases Has a Similar Response to Mat Pilates Training
Source: Antioxidants (Basel). 2022 Jul 26;11(8):1445. doi: 10.3390/antiox11081445 (PMC9331979; doi:10.3390/antiox11081445)
Supplement: Supplementary file 1 [file antioxidants-11-01445-s001.zip › antioxidants-1746032-supplementary.pdf]

antioxidants

MDPI

Supplementary Materials

Redox status of postmenopausal women with single or multiple cardiometabolic diseases has a similar response to Mat Pilates training

Supplementary Materials:

Supplementary Table S1: Mat Pilates exercise program.

Exercise	Description
	**Warm up**
Roll up	Dorsal decubitus, arms going toward the shoulder line, and from there the head starts to rise and the trunk curls towards the feet.
**Knee folds**	Lie on your back with your pelvis in neutral position, with your knees bent and feet flat. Exhale and tighten your abs to lift your right leg straight up to 90 degrees. Inhale, then exhale and lift your left leg straight up to 90 degrees. Lower your legs to the floor.
**Knee sways**	Lying in a dorsal decubitus with a bend in your knees and your feet relaxed flat on the surface. Keep your knees and feet lightly touching and your shoulders flat against the surface as you slowly sway your knees together from side to side.
	**Training A**
**The one leg stretch**	Neutral position lying on back, slowly raise one leg and bend it, forming a 90 ° angle, without leaving the neutral position. Extend the leg forward taking care not to arch your back and raise the other leg extending to which it was folded but without lowering the leg.
One leg circle (right / left)	Dorsal decubitus, straight legs and circular movements with both legs, one at a time clockwise.
One leg circle (right / left)	Dorsal decubitus, straight legs and circular movements with both legs, one at a time in the counterclockwise direction.
**The shoulder bridge in the ball**	Lying face up, bend the knees and place the feet on top of a stability ball. Rest on the shoulders and the upper back while keeping the body in a straight line from the knees to the head. Slowly lower the hips back down to the floor, keeping the ball still.
The Swan dive	Ventral decubitus, palms resting on the floor, elbows extended. Keep the spine extension, triggered abs, buttocks and contractors and shoulders away from the ears as the body swings upward and returns toward the floor.
Abdominal with the ball	Contraction of the abdominal muscle, leave the hip on the floor and support both legs on top of the ball, with the hands resting on the chest, remove the shoulder blades from the floor and return to the starting position.

The Hundred with flex	Lying with the backrest on the mat, head and torso slightly raised, legs raised and arms stretched to the side of the hip without touching the floor. Inhale to raise the head and trunk until reaching the base of the scapula, maintaining control of the body and returning to the initial position. The flex ring should be held between the ankles.
Abdominal with the ball (alternating legs)	Abdominal muscle contraction, leave the hips on the floor and support both legs at the top of the ball, with the hands resting behind the neck, withdraw the shoulder blades from the floor going with the right elbow towards the left knee that will leave the ball support and return to the starting position to repeat the exercise with the left elbow toward the right knee.
**Training B**	
The double leg stretch	Neutral position lying on your back, slowly lift your legs and bend them at a 90° angle, without leaving the neutral position. Extend your legs forward taking care not to arch but without letting your legs touch the floor.
The Shoulder Bridge	Lying face up, bend the knees and place the feet on the floor. Rest on the shoulders and the upper back while keeping the body in a straight line from the knees to the head. Slowly raise and lower hips back to the floor.
The Shoulder Bridge with one leg (right / left) and flex	Lying straight up, bend your knees and extend one leg toward the ceiling. Rest on the shoulders and upper back, keeping the body straight from the knees to the head. Slowly raise and lower your hip back to the floor. Repeat this movement with the other leg. Hold the flex ring at chest height with your elbows bent at shoulder height and tighten every time your hips are raised.
The shoulder bridge with flex ring	Lying face up, bend the knees and place the feet on the floor. Rest on the shoulders and the upper back while keeping the body in a straight line from the knees to the head. Slowly lower the hips back down to the floor. Position the flex ring between the inner thigh and press every time you lift your hip from the floor.
Swimming	Neutral position facing down, raise arms and place forward for a V with shoulders. The lumbar area should be stable, without letting the belly yield towards the floor, but keeping the abdomen contracted. Lift both the right arm and the left leg simultaneously at the same height. Lower your right arm and left leg at the same time. Repeat the exercise using the left arm and right leg this time.
The Swan dive in the ball	Ventral decubitus on top of the ball, palms resting on the floor, elbows extended. Hold the extension of the spine, trigger abs, buttocks, contractors and shoulders away from the ears as the body moves up and returns to the ball.
The Hundred	Lying with the backrest on the mat, head and torso slightly raised, legs raised and arms stretched to the side of the hip without touching the floor. Inhale to raise the head and trunk until reaching the base of the scapula, maintaining control of the body and returning to the initial position.
Board	Lean forward, lean on the elbows in the floor aligned with the shoulder.

	Then raise the hip, keeping only supported by the tip of the feet and the elbows. Exercise has no movement, characterizing isometry.
**Cooling down**	
Spine Stretch	Sitting with the spine straight, legs stretched and open slightly beyond the width of the hips. The arms remain stretched forward, always maintaining the alignment of the shoulder blades. It takes the chin to the chest and rolls the column forward, forming a "C". The chest should pass over the thigh and the inner part of the thighs should be lengthening. Return the movement to the starting position.
Cat Stretch	In four supports, with hands under the shoulders, the knees under the hips and the legs apart in the distance of the hips. Pelvis and spine in the neutral position. The head should follow the alignment of the thoracic spine. The movement is to articulate the spine, without misaligning the hands and legs. Tilt posteriorly to the pelvis and round the spine, sequentially joining the coccyx to the head. Maintain the position with the abs contracted and supporting the head with relaxed shoulders. We rejoined the spine sequentially to an extension of the thoracic.
Roll down	Stand your feet shoulder-width apart. The column must be in a neutral position. Lower the body, rolling it forward, keeping the muscles of the neck and arms totally relaxed. Try touching the shins or feet with your hands. Slowly return to the starting position.

Supplementary Table S2: Analysis of food consumption pattern in SINGLE (n=13) and MULT (n=20) before and after 12 weeks of Mat Pilates training.

	PRE Mean ± SD	POST adjusted Mean ± SD	Difference in means (CI 95%)	ANCOVA p
**Energy (kcal)**				
SINGLE	1608.19 ± 499.62	1597.94 ± 355.35	-10.25 (-706.92 to 686.42)	0.847
MULT	1649.27 ± 603.15	1622.59 ± 440.76	-26.68 (-214.02 to 160.66)	
**Protein (g)**				
SINGLE	61.37 ± 13.88	59.55 ± 19.22	-1.82 (-10.93 to 7.29)	0.472
MULT	74.36 ± 36.08	64.58 ± 19.12	-9.78 (-22.43 to 2.87)	
**Carbohydrate (g)**				
SINGLE	186.37 ± 54.06	188.11 ± 48.29	1.74 (-26.12 to 29.6)	0.790
MULT	187.57 ± 50.74	192.73 ± 48.29	5.16 (-16.55 to 26.87)	
**Lipids (g)**				
SINGLE	69.51 ± 29.14	68.86 ± 21.33	-0.65 (-14.53 to 13.23)	0.661
MULT	76.41 ± 38.06	65.48 ± 21.31	-10.93 (-24.45 to 2.59)	
**Saturated (g)**				
SINGLE	22.74 ± 11.98	19.05 ± 7.02	-3.69 (-9.03 to 1.65)	0.980
MULT	21.13 ± 8.71	18.99 ± 7.02	-2.14 (-5.61 to 1.33)	
**Monounsaturated (g)**				

SINGLE	19.26 ± 8.32	17.47 ± 6.73	-1.79 (-5.9 to 2.32)	0.599
MULT	20.71 ± 9.78	18.74 ± 6.73	-1.97 (-5.65 to 1.71)	
**Polyunsaturated (g)**				
SINGLE	18.11 ± 7.27	19.27 ± 6.55	1.16 (-2.6 to 4.92)	0.538
MULT	19.78 ± 7.73	17.81 ± 6.55	-1.97 (-5.11 to 1.17)	
**Cholesterol (mg)**				
SINGLE	279.27 ± 149.16	220.92 ± 84.95	-58.35 (-124.33 to 7.63)	0.800
MULT	243.65 ± 118.05	213.14 ± 84.78	-30.51 (-75.55 to 14.53)	
**Fiber (g)**				
SINGLE	15.69 ± 4.36	15.64 ± 4.26	-0.05 (-2.39 to 2.29)	0.967
MULT	15.36 ± 5.03	15.58 ± 4.26	0.22 (-1.82 to 2.26)	
**Zinc (mg)**				
SINGLE	6.57 ± 2.20	6.96 ± 3.16	0.39 (-1.09 to 1.87)	0.533
MULT	8.72 ± 5.70	7.68 ± 3.14	-1.04 (-3.06 to 0.98)	
**Vitamin A (µg)**				
SINGLE	115.7 ± 76.05	116.13 ± 41.97	0.43 (-32.96 to 33.82)	0.007
MULT	94.66 ± 60.47	72.72 ± 41.86	-21.94 (-44.73 to 0.85)	
**Vitamin C (mg)**				
SINGLE	176.24 ± 96.71	113.31 ± 86.78	-62.93 (-112.88 to -12.98)	0.510
MULT	125.74 ± 95.48	134.20 ± 107.64	8.46 (-21.22 to 38.14)	

Data presented as mean ± standard deviation. SINGLE: women with 1 disease; MULT: women with ≥ 2 comorbidities. Data presented in pre- and post-adjusted. Variation values and confidence intervals were calculated from the unadjusted post.
